# Berg Balance Scale in post-COVID-19 patients: results from a retrospective study

**DOI:** 10.25122/jml-2025-0098

**Published:** 2025-06

**Authors:** Ovidiu Cristian Chiriac, Daniela Miricescu, Corina Sporea, Ana Raluca Mitrea, Silviu Stanciu, Raluca Mititelu, Raluca Grigore, Sarah Adriana Nica

**Affiliations:** 1 Discipline of Balneophysiokinetotherapy and Recovery, Faculty of Midwifery and Nursing, Carol Davila University of Medicine and Pharmacy, Bucharest, Romania; 2 Dr. Carol Davila Central Military Emergency University Hospital, Bucharest, Romania; 3 Discipline of Biochemistry, Faculty of Dentistry, Carol Davila University of Medicine and Pharmacy, Bucharest, Romania; 4 Dr. Nicolae Robănescu National University Center for Children’s Neurorehabilitation, Bucharest, Romania; 5 Physical Medicine and Rehabilitation (Medical Recovery Neurology), Carol Davila University of Medicine and Pharmacy, Bucharest, Romania; 6 Department of Internal Medicine and Gastroenterology, Central Military Emergency University Hospital, Carol Davila University of Medicine and Pharmacy, Bucharest, Romania; 7 Department of Nuclear Medicine, University of Medicine and Pharmacy Carol Davila, Bucharest, Romania; 8 Clinic of Nuclear Medicine, Dr Carol Davila Central University Emergency Military Hospital, Bucharest, Romania; 9 ENT, Head & Neck Surgery Department, Coltea Clinical Hospital, Carol Davila University of Medicine and Pharmacy, Bucharest, Romania; 10 Department of Physical Medicine and Rehabilitation, Carol Davila University of Medicine and Pharmacy, Bucharest, Romania

**Keywords:** viral infection, isolation, sedentarism, muscle weakness

## Abstract

SARS-CoV-2 infection has been linked to sedentary behavior, which can lead to musculoskeletal weakness, cardiometabolic disorders, and a worsening of pre-existing conditions, particularly in older patients. The primary aim of our study was to compare the Berg Balance Score (BBS), comprising BBS1 (sit to stand), BBS2 (standing unsupported), and BBS3 (sitting unsupported), in patients with mild and moderate post-COVID-19 symptoms before and after the recovery program. BBS1, BBS2, and BBS3 demonstrated statistically significant improvement after the recovery program for both men and women compared to the initial assessment (*P* < 0.001). In terms of age, BBS1, BBS2, and BBS3 were significantly higher in patients over 60 years old (*P* < 0.001). Statistically significant differences were observed between BBS1 in patients over 60 and those under 60 years (*P* = 0.008). Significant negative correlations were found between age and BBS1 (*P* = 0.001; R = -0.267). Significant positive correlations were noted between BBS1 and BBS2 (*P* < 0.001; R = 0.827), BBS1 and BBS3 (*P* < 0.001; R = 0.796), and BBS2 and BBS3 (*P* < 0.001; R = 0.926). The recovery program implemented for post-COVID-19 patients significantly improved BBS subitems, positively impacting motor function.

## INTRODUCTION

Severe acute respiratory syndrome coronavirus 2 (SARS-CoV-2) is the etiological agent of coronavirus disease 2019 (COVID-19), a highly contagious disease primarily transmitted through respiratory droplets generated by coughing, sneezing, or close interpersonal contact [1]. SARS-CoV-2 was identified in humans in 2003 as a betacoronavirus. Since its discovery, thousands of coronaviruses have been recognized, including alpha, beta, gamma, and delta-CoV [2].

The disease presents a variety of symptoms, ranging from asymptomatic cases to severe complications. As a result, patients may experience respiratory, gastrointestinal, or cardiac issues. Additionally, ophthalmic and gustatory dysfunctions, such as anosmia and ageusia, frequently occur, indicating the loss of smell and taste [3]. RT-PCR is the gold standard method for identifying SARS-CoV-2 RNA. A decreased viral load can sometimes result in false-negative test results [4].

Beyond the acute infection, COVID-19 has led to profound disruptions in lifestyle. Lockdowns, quarantine, and isolation resulted in increased sedentary behavior, particularly in elderly individuals, exacerbating musculoskeletal weakness, sarcopenia, and cardiometabolic conditions and worsening pre-existing comorbidities [5]. The COVID-19 pandemic also affected children as they were forced to stay home, leading to various disruptions that impacted their health and well-being [6].

Among the many areas affected in post-COVID-19 patients, balance deficits pose a clinically significant issue with both short- and long-term consequences. Balance, a composite function that involves sensory integration, musculoskeletal integrity, and central processing, is particularly vulnerable to systemic inflammation, prolonged immobility, and multisystem involvement—all characteristic features of post-viral syndromes like COVID-19. Impaired balance affects patients’ independence, increases the risk of falls, hinders reintegration into daily life, and adversely impacts overall quality of life. Therefore, evaluating and addressing balance in post-COVID patients has become a priority in physical medicine and rehabilitation [7,8].

In clinical practice, the Berg Balance Scale (BBS) is one of the most widely used standardized tools for objectively assessing static and dynamic balance. It consists of 14 items that evaluate various functional tasks, including transfers, standing, reaching, turning, and postural transitions, providing a detailed profile of a patient’s balance capacity. While the total score is typically used to quantify fall risk and inform therapeutic decisions, analyzing performance on individual BBS subitems can offer deeper insights into specific postural control deficits. This is particularly important for patients with low initial functionality; the early subitems of the BBS, such as sit-to-stand (BBS1), standing unsupported (BBS2), and sitting unsupported (BBS3), are essential for tracking minimal yet meaningful changes in basic mobility [7-9].

In the context of post-COVID-19 rehabilitation, it is crucial to explore whether patients improve functionally and how specific dimensions of balance evolve with demographic variables such as age and sex. Advanced age has long been linked to impaired balance and diminished recovery potential; however, little is known about how post-COVID patients of varying ages respond to rehabilitation concerning core postural abilities. Examining these factors can contribute to a more individualized rehabilitation approach and help identify patients at risk of poor outcomes.

This study examined a comparative analysis of BBS subitem scores in a post-COVID patient cohort undergoing inpatient rehabilitation. The primary objective was to assess the evolution of static balance abilities—specifically, the tasks related to BBS1 (sit-to-stand), BBS2 (standing unsupported), and BBS3 (sitting unsupported)—in post-COVID-19 patients who began the recovery program in the hospital and continued at home until new readmission. A secondary objective was to investigate whether improvements in these balance subcomponents differed significantly by age group (under 60 versus 60 years and older) or sex (men versus women).

## MATERIAL AND METHODS

### Study design and participants

This retrospective study included 160 patients (97 women and 74 men) diagnosed with mild to moderate COVID-19 infection at the Dr. Carol Davila Military Emergency Hospital. BBS outlines specific actions that the patient must perform. All patients participated in a structured recovery program and were evaluated using the Berg Balance Scale (BBS) at two-time points: during initial hospital admission and upon readmission following a period of home-based rehabilitation.

### Berg Balance Scale (BBS) assessment

The BBS is a standardized clinical tool composed of 14 functional tasks, each rated on a scale from 0 to 4 (0 = unable to perform; 4 = able to perform independently without difficulty). For this study, particular focus was placed on three subitems: BBS1 (sit to stand), BBS2 (standing unsupported), and BBS3 (sitting unsupported). These subitems were used to classify patients into clinical-functional stages based on their performance and level of cooperation, evaluated using the S5Q scale.

Level 0 indicated a failure to meet the basic assessment criteria along with an uncooperative attitude (level 0 on the S5Q scale). Level 1 included patients who were cooperative, partially cooperative, or uncooperative (S5Q score of 1 to 5) but were not permitted to perform transfers from a lying to a sitting or standing position. Level 2 included cooperative patients (S5Q score of 3 to 5) who met the basic assessment criteria but were unable to perform transfers actively. Level 3 corresponded to cooperative patients (S5Q score of 4 or 5) with a Medical Research Council (MRC) score above 36, BBS1 and BBS2 scores of 0, and a BBS3 score of at least 1. Level 4 applied to fully cooperative individuals (S5Q score of 5) with MRC scores above 48, a BBS1 score of 0, a BBS2 score greater than 0, and a BBS3 score greater than 2. Level 5 described fully cooperative patients (S5Q score of 5) who met the same MRC criteria and achieved BBS1 scores above 1, BBS2 above 2, and BBS3 above 3.

### Rehabilitation program

The medical recovery program for patients with mild to moderate COVID-19 included light physical exercises at bed level to stimulate muscle tone in both the upper and lower limbs, respiratory exercises, trunk movements to encourage standing and walking, and cognitive training techniques. Verticalization was introduced gradually, beginning with supported short sitting at the edge of the bed and progressing to assisted ambulation under the supervision of trained rehabilitation personnel. After discharge, patients continued a home-based recovery program. They were encouraged to maintain vertical posture, engage in progressive walking, and perform low-intensity aerobic activities to improve functional independence. The rehabilitation regimen also included occupational therapy interventions aimed at restoring and training activities of daily living (ADLs), as prescribed by a medical rehabilitation physician.

### Statistical analysis

Data were analyzed using IBM SPSS Statistics version 25 and visualized with Microsoft Office Excel and Word 2024. Quantitative variables were reported as means with standard deviations or as medians with interquartile ranges. The normality of the quantitative variables was assessed using the Shapiro-Wilk test. Quantitative independent variables with non-parametric distributions were tested between groups using Mann-Whitney U tests or Kruskal-Wallis H tests, accompanied by post-hoc Dunn-Bonferroni tests. Correlations between these variables were estimated with Spearman’s rho correlation coefficient. Quantitative independent variables with normal distributions were tested between groups using Student’s *t*-test after evaluating the equality of variances with Levene’s test. For quantitative paired variables with non-parametric distributions, Wilcoxon’s tests were used for measurements. Qualitative variables were reported as counts or percentages. Qualitative independent variables were tested between groups using the Pearson Chi-Square Test or Fisher’s Exact Test. Z-tests with Bonferroni correction clarified the results obtained from the contingency tables. Qualitative paired variables were tested between measurements using the Related-Samples Marginal Homogeneity test, with post-hoc Related-Samples McNemar tests, where the significance threshold was adjusted to 0.0167 with a Bonferroni correction. The significance level for all tests was set at α = 0.05.

## RESULTS

Data from Table 1 compares BBS1 scores at baseline and after recovery. The distribution of BBS1 scores was non-parametric at both time points, as confirmed by the Shapiro-Wilk test (*P* < 0.05). BBS1 was significantly higher after recovery (5^th^ percentile = 0, 95^th^ percentile = 2 vs. 5^th^ percentile = 0, 95^th^ percentile = 3, *P* <0.001), indicating a notable increase of 0.36 ± 0.74 points (median = 0, IQR = 0–0).

**Table 1:** Comparison of BBS1 before and after recovery

BBS1	Mean ± SD	Median (IQR)	P*
Initial (*P* < 0.001**)	0.58 ± 0.96	E0 (0–2)	< 0.001
After recovery (*P* < 0.001**)	0.94 ± 1.15	0 (0–2)	

* Related-Samples Wilcoxon Signed Rank Test, **Shapiro-Wilk Test

Data from Table 2 compares BBS2 scores between the initial measurement and the recovery period. The distribution of the variable was non-parametric for both measurements, as indicated by the Shapiro-Wilk test (*P* < 0.05). BBS2 was significantly higher after recovery (median = 2, IQR = 1–3 vs. median = 1, IQR = 0–3, *P* < 0.001), reflecting a notable increase of 0.73 ± 0.82 points (median = 1, IQR = 0–1).

**Table 2:** Comparison of BBS2 between the initial measurement and post-recovery

BBS2	Mean ± SD	Median (IQR)	P*
Initial (*P* < 0.001**)	1.14 ± 1.28	1 (0–3)	< 0.001
After recovery (*P* < 0.001**)	1.88 ± 1.16	2 (1–3)	

* Related-Samples Wilcoxon Signed Rank Test, **Shapiro-Wilk Test

Data from Table 3 compares BBS3 between the initial measurement and after recovery. The distribution of the scores was non-parametric for both measurements, as indicated by the Shapiro-Wilk test (*P* < 0.05). BBS3 was significantly higher after recovery (median = 3, IQR = 3–4 vs. median = 3, IQR = 2–4, *P* < 0.001), showing a significant increase of 0.67 ± 0.84 points (median = 0, IQR = 0–1).

**Table 3:** Comparison of BBS3 between the initial measurement and post-recovery

BBS3	Mean ± SD	Median (IQR)	P*
Initial (*P* < 0.001**)	2.56 ± 1.13	3 (2–4)	< 0.001
After recovery (*P* < 0.001**)	3.23 ± 0.81	3 (3–4)	

* Related-Samples Wilcoxon Signed Rank Test, **Shapiro-Wilk Test

Data from Table 4 compares BBS1 scores among women before and after recovery. The distribution of the variable was non-parametric for both measurements, according to the Shapiro-Wilk test (*P* < 0.05). BBS1 was significantly higher after recovery (5^th^ percentile = 0, 95^th^ percentile = 2 vs. 5^th^ percentile = 0, 95^th^ percentile = 3, *P* < 0.001), showing a notable increase of 0.39 ± 0.78 points (median = 0, IQR = 0–1).

**Table 4:** BBS1 scores among women before and after recovery

BBS1	Mean ± SD	Median (IQR)	P*
Initial (*P* < 0.001**)	0.65 ± 1.03	0 (0–2)	< 0.001
After recovery (*P* < 0.001**)	1.04 ± 1.19	0 (0–2)	

* Related-Samples Wilcoxon Signed Rank Test, **Shapiro-Wilk Test

Data from Table 5 compares BBS2 scores among women before and after recovery. The distribution of the variable was non-parametric in both measurements, according to the Shapiro-Wilk test (*P* < 0.05). BBS2 was significantly higher after recovery (median = 2, IQR = 1–3 vs. median = 1, IQR = 0–3, *P* < 0.001), with a notable increase of 0.7 ± 0.88 points (median = 1, IQR = 0–1).

**Table 5:** BBS2 scores among women before and after recovery

BBS2	Mean ± SD	Median (IQR)	P*
Initial (*P* < 0.001**)	1.22 ± 1.31	1 (0–3)	<0.001
After recovery (*P* < 0.001**)	1.92 ± 1.21	2 (1–3)	

* Related-Samples Wilcoxon Signed Rank Test, **Shapiro-Wilk Test

Data from Table 6 compares BBS3 scores in women before and after recovery. The distribution of the variable was non-parametric in both measurements, as indicated by the Shapiro-Wilk test (*P* < 0.05). BBS3 significantly increased after recovery (median = 3, IQR = 3–4 vs. median = 3, IQR = 2–4, *P* < 0.001), with a notable increase of 0.58 ± 0.84 points (median = 0, IQR = 0–1).

**Table 6:** BBS3 scores among women before and after recovery

BBS3	Mean ± SD	Median (IQR)	P*
Initial (*P* < 0.001**)	2.62 ± 1.14	3 (2–4)	<0.001
After recovery (*P* < 0.001**)	3.2 ± 0.92	3 (3–4)	

* Related-Samples Wilcoxon Signed Rank Test, **Shapiro-Wilk Test

Data from Table 7 compares BBS1 scores in men between the initial measurement and post-recovery. The distribution of the variable was non-parametric for both measurements, as indicated by the Shapiro-Wilk test (*P* < 0.05). BBS1 was significantly higher after recovery (median = 0, IQR = 0–2 vs. median = 0, IQR = 0–0, *P* = 0.001), showing a significant increase of 0.32 ± 0.69 points (median = 0, IQR = 0–0).

**Table 7:** BBS1 scores among men before and after recovery

BBS1	Mean ± SD	Median (IQR)	P*
Initial (*P* < 0.001**)	0.48 ± 0.86	0 (0–0)	0.001
After recovery (*P* < 0.001**)	0.79 ± 1.08	0 (0–2)	

* Related-Samples Wilcoxon Signed Rank Test, **Shapiro-Wilk Test

Data from Table 8 compares BBS2 scores in men between the initial measurement and after recovery. The distribution of the variable was non-parametric for both measurements, according to the Shapiro-Wilk test (*P* < 0.05). BBS2 was significantly higher following recovery (median = 1, IQR = 1–3 compared to median = 1, IQR = 0–2, *P* = 0.001), showing an average increase of 0.78 ± 0.72 points (median = 1, IQR = 0–1).

**Table 8:** BBS2 scores among men before and after recovery

BBS2	Mean ± SD	Median (IQR)	P*
Initial (*P* < 0.001**)	1.03 ± 1.23	1 (0–2)	<0.001
After recovery (*P* < 0.001**)	1.81 ± 1.07	1 (1–3)	

* Related-Samples Wilcoxon Signed Rank Test, **Shapiro-Wilk Test

Data from Table 9 compares BBS3 scores in men before and following recovery. The distribution of the variable was non-parametric in both measurements, as indicated by the Shapiro-Wilk test (*P* < 0.05). BBS3 was significantly higher after recovery (median = 3, IQR = 3–4 vs. median = 2, IQR = 1–3, *P* = 0.001), showing a notable increase of 0.81 ± 0.84 points (median = 1, IQR = 0–2).

**Table 9:** BBS3 scores among men before and after recovery

BBS3	Mean ± SD	Median (IQR)	P*
Initial (*P* < 0.001**)	2.48 ± 1.12	2 (1–3)	<0.001
After recovery (*P* < 0.001**)	3.29 ± 0.63	3 (3–4)	

* Related-Samples Wilcoxon Signed Rank Test, **Shapiro-Wilk Test

Table 10 compares BBS1 scores before and after recovery in patients aged 60 years and older. The distribution of the variable was non-parametric in both sets of measurements, as indicated by the Shapiro-Wilk test (*P* < 0.05). BBS1 was significantly higher after recovery (median = 0, IQR = 0–2 vs. median = 0, IQR = 0–0, p=0.014), reflecting a notable increase of 0.22 ± 0.68 points (median = 0, IQR = 0–0).

**Table 10:** BBS1 scores in patients aged ≥ 60 before and after recovery

BBS1	Mean ± SD	Median (IQR)	P*
Initial (*P* < 0.001**)	0.34 ± 0.84	0 (0–0)	0.014
After recovery (*P* < 0.001**)	0.55 ± 0.96	0 (0–2)	

* Related-Samples Wilcoxon Signed Rank Test, **Shapiro-Wilk Test

Table 11 compares BBS2 scores in patients aged 60 years and older before and after recovery. The distribution of the variable was non-parametric in both measurements according to the Shapiro-Wilk test (*P* < 0.05). BBS2 was significantly higher after recovery (median = 1, IQR = 1–3 vs. median = 1, IQR = 0–2, *P* < 0.001), with a notable increase of 0.76 ± 0.84 points (median = 1, IQR = 0–1).

**Table 11:** BBS2 scores in patients aged ≥ 60 before and after recovery

BBS2	Mean ± SD	Median (IQR)	P*
Initial (*P* < 0.001**)	0.7 ± 1.1	0 (0–1)	<0.001
After recovery (*P* < 0.001**)	1.46 ± 1.11	1 (1–3)	

* Related-Samples Wilcoxon Signed Rank Test, **Shapiro-Wilk Test

Table 12 compares BBS3 scores in patients aged 60 years and older before and after recovery. The distribution of the variable was non-parametric in both measurements, according to the Shapiro-Wilk test (*P* < 0.05). Post-recovery, BBS3 was significantly higher (median = 3, IQR = 2–4 vs. median = 2, IQR = 1–3, *P* < 0.001), reflecting a significant increase of 0.73 ± 0.89 points (median = 1, IQR = 0–1).

**Table 12:** BBS3 scores in patients aged ≥ 60 before and after recovery

BBS3	Mean ± SD	Median (IQR)	P*
Initial (*P* < 0.001**)	2.19 ± 1.03	2 (1–3)	<0.001
After recovery (*P* < 0.001**)	2.92 ± 0.88	3 (2–4)	

* Related-Samples Wilcoxon Signed Rank Test, **Shapiro-Wilk Test

Table 13 compares the difference in BBS1 scores between the initial and post-recovery measurements according to gender. The distribution of the difference was non-parametric in both groups, as indicated by the Shapiro-Wilk test (*P* < 0.05). Differences between groups were not significant (*P* = 0.425); therefore, the evolution of BBS1 was not substantial by gender.

**Table 13:** BBS1 change by gender following recovery

Gender	Mean ± SD	Median (IQR)	Mean Rank	P*
Woman (*P* < 0.001**)	0.39 ± 0.78	0 (0–1)	82.25	0.425
Man (*P* < 0.001**)	0.32 ± 0.69	0 (0–0)	77.80	

* Mann-Whitney U Test, **Shapiro-Wilk Test

Data from Table 14 compares BBS2 scores between initial and post-recovery measurements by gender. The distribution of the difference was non-parametric in both groups, as indicated by the Shapiro-Wilk test (*P* < 0.05). The differences between groups were not significant (*P* = 0.263); therefore, the evolution of BBS2 was not significantly different by gender.

**Table 14:** BBS2 change by gender following recovery

Gender	Mean ± SD	Median (IQR)	Mean Rank	P*
Woman (*P* < 0.001**)	0.7 ± 0.88	1 (0–1)	77.46	0.263
Man (*P* < 0.001**)	0.78 ± 0.72	1 (0–1)	85.17	

* Mann-Whitney U Test, **Shapiro-Wilk Test

Table 15 presents a comparison of BBS2 score improvements between male and female participants. The distribution of BBS2 difference scores was non-parametric in both groups, as confirmed by the Shapiro-Wilk test (*P* < 0.05). Although both groups demonstrated significant improvements from baseline, the between-group difference was not statistically significant (*P =* 0.263).

**Table 15:** BBS3 change by gender following recovery

Gender	Mean ± SD	Median (IQR)	Mean Rank	P*
Woman (*P* < 0.001**)	0.58 ± 0.84	0 (0–1)	75.44	0.063
Man (*P* < 0.001**)	0.81 ± 0.84	1 (0–2)	88.29	

* Mann-Whitney U Test, **Shapiro-Wilk Test

Table 16 presents a comparison of the change in BBS1 scores between initial and post-recovery assessments, stratified by age group. The distribution of score differences was non-parametric in both groups, as confirmed by the Shapiro-Wilk test (*P* < 0.05). A statistically significant difference was observed between age groups (*P =* 0.008, Mann-Whitney U Test). Patients younger than 60 years had a greater improvement in BBS1 scores (median = 0, IQR = 0–1) compared to those aged 60 years or older (median = 0, IQR = 0–0).

**Table 16:** BBS1 score improvement by age group

Age	Mean ± SD	Median (IQR)	Mean Rank	P*
< 60 years (*P* < 0.001**)	0.49 ± 0.78	0 (0–1)	86.71	0.008
≥ 60 years (*P* < 0.001**)	0.22 ± 0.68	0 (0–0)	72.30	

* Mann-Whitney U Test, **Shapiro-Wilk Test

Table 17 illustrates the comparison of BBS2 score improvements between initial and post-recovery assessments, stratified by age group. The distribution of score differences was non-parametric for both groups, as confirmed by the Shapiro-Wilk test (*P* < 0.05). Although both age groups had significant within-group improvement in BBS2 scores, the between-group difference was not statistically significant (*P =* 0.616), suggesting that age did not significantly affect the degree of improvement in standing balance following recovery.

**Table 17:** BBS2 score improvement by age group

Age	Mean ± SD	Median (IQR)	Mean Rank	P*
< 60 years (*P* < 0.001**)	0.71 ± 0.81	1 (0-1)	78.43	0.616
≥ 60 years (*P* < 0.001**)	0.76 ± 0.84	1 (0-1)	81.80	

* Mann-Whitney U Test, **Shapiro-Wilk Test

Data from Table 18 compares BBS3 differences between initial and post-recovery measurements by age. The distribution of the difference was non-parametric in both groups according to the Shapiro-Wilk test (*P* < 0.05). Differences between groups were not significant (*P* = 0.346); therefore, the evolution of BBS3 was not significant by age.

**Table 18:** BBS3 score improvement by age group

Age	Mean ± SD	Median (IQR)	Mean Rank	P*
< 60 years (*P* < 0.001**)	0.61 ± 0.8	0 (0-1)	77.04	0.346
≥ 60 years (*P* < 0.001**)	0.73 ± 0.89	1 (0-1)	83.41	

* Mann-Whitney U Test, **Shapiro-Wilk Test

**Figure 1:**
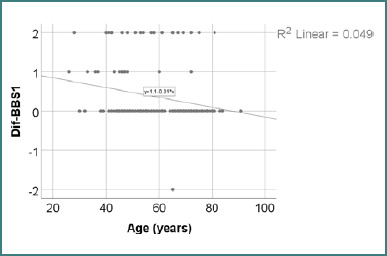
Correlation between age and changes in BBS1 score

Data from Table 19 and Figure 1 demonstrate the correlation between age and the change in BBS1 scores. The distribution of BBS1 difference scores was non-parametric, as confirmed by the Shapiro-Wilk test (*P* < 0.05). The observed correlation was significant, negative, and of moderate strength (*P* = 0.001, R = -0.267), indicating that younger patients were significantly associated with a greater increase in BBS2 after recovery, while older patients experienced the opposite effect.

**Table 19:** Correlation between age and changes in BBS1 score

Correlation	P*
Age (*P* = 0.033**) x BBS1 (*P* < 0.001**)	0.001, R = -0.267

* Spearman’s rho Correlation Coefficient, **Shapiro-Wilk Test

**Figure 2:**
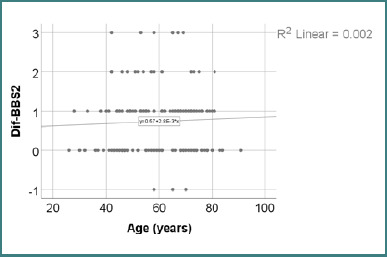
Correlation between age and changes in BBS2 score

Data from Table 20 and Figure 2 indicate the correlation between age and the changes in BBS2 scores. According to the Shapiro-Wilk test (*P* < 0.05), the distribution of the BBS2 difference scores was non-parametric. The observed correlation was not significant (*P* = 0.461); thus, no significant correlation was found between patient age and the evolution of BBS2 scores.

**Table 20:** Correlation between age and changes in BBS2 score

Correlation	P*
Age (*P* = 0.033**) x BBS2 (*P* < 0.001**)	0.461, R = 0.059

* Spearman’s rho Correlation Coefficient, **Shapiro-Wilk Test

**Figure 3:**
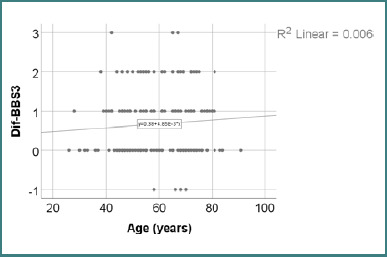
Correlation between age and changes in BBS3 score

Table 21 and Figure 3 present the correlation between age and the change in BBS3 scores following recovery. The distribution of BBS3 difference scores was non-parametric, as confirmed by the Shapiro-Wilk test (*P* < 0.05). The analysis revealed no statistically significant correlation between age and BBS3 improvement (*P* = 0.249), indicating that patient age was not associated with the degree of change in sitting balance following the recovery program.

**Table 21:** Correlation between age and changes in BBS3 score

Correlation	P*
Age (*P* = 0.033**) x BBS3 (*P* < 0.001**)	0.249, R = 0.092

* Spearman’s rho Correlation Coefficient, **Shapiro-Wilk Test

**Figure 4:**
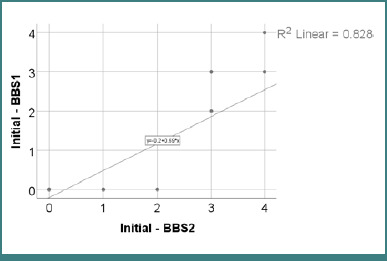
Correlation between initial BBS1 and BBS2 scores

Data from Table 22 and Figure 4 illustrate the correlation between initial BBS1 and BBS2 scores. The distribution of both variables was non-parametric, as indicated by the Shapiro-Wilk test (*P* < 0.05). The observed correlation was significant and positive, with very high power (*P* < 0.001, R = 0.827). Patients with higher BBS1 scores were significantly more likely to have higher BBS2 scores, and vice versa.

**Table 22:** Correlation between initial BBS1 and BBS2 scores

Correlation	P*
BBS1 (*P* < 0.001**) x BBS2 (*P* < 0.001**)	<0.001, R = 0.827

* Spearman’s rho Correlation Coefficient, **Shapiro-Wilk Test

**Figure 5:**
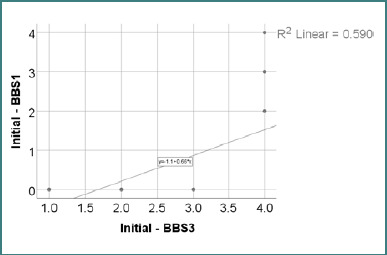
Correlation between initial BBS1 and BBS3 scores

Data from Table 23 and Figure 5 demonstrate the relationship between initial BBS1 and BBS3 scores. The distribution of both variables was non-parametric, as indicated by the Shapiro-Wilk test (*P* < 0.05). The observed correlation was both significant and positive, with very high power (*P* < 0.001, R = 0.796); patients with higher BBS1 scores were significantly more likely to have higher BBS3 scores and vice versa.

**Table 23:** Correlation between initial BBS1 and BBS3 scores

Correlation	P*
BBS1 (*P* < 0.001**) x BBS3 (*P* < 0.001**)	<0.001, R = 0.796

* Spearman’s rho Correlation Coefficient, **Shapiro-Wilk Test

**Figure 6:**
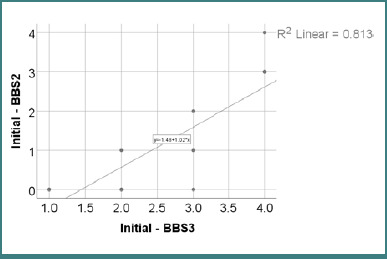
Correlation between initial BBS2 and BBS3 scores

Data from Table 24 and Figure 6 illustrate the correlation between initial BBS2 and BBS3 scores. The distribution of both variables was non-parametric, as determined by the Shapiro-Wilk test (*P* < 0.05). The observed correlation was significant and positive, showing very high power (*P* < 0.001, R = 0.926); patients with higher BBS2 scores were significantly more likely to have higher BBS3 scores, and vice versa.

**Table 24:** Correlation between initial BBS2 and BBS3 scores

Correlation	P*
BBS2 (*P* < 0.001**) x BBS3 (*P* < 0.001**)	<0.001, R = 0.926

* Spearman’s rho Correlation Coefficient, **Shapiro-Wilk Test

## DISCUSSION

The COVID-19 pandemic has significantly affected not only global healthcare systems but also the physical condition of millions of survivors who continue to face persistent functional limitations well beyond the acute phase [10]. Increasingly, attention has shifted to the subacute and long-term consequences of COVID-19, particularly regarding neuromuscular coordination [11], postural control [12,13], and overall physical deconditioning [14,15]. Fatigue [16,17], dyspnea [18,19], orthostatic intolerance [20-22], balance impairments [23-26], and muscle weakness [27-29] are commonly reported among individuals recovering from moderate to severe SARS-CoV-2 infection, necessitating targeted rehabilitation strategies aimed at restoring functional autonomy.

The BBS scale is widely used to assess balance performance and evaluate fall risk in older adults. It consists of 14 items in which participants must maintain their balance across various tasks and positions, each presenting different levels of difficulty [30]. The BBS scale is also utilized to evaluate patients diagnosed with other conditions, such as stroke [31]. However, research focusing on the use of the BBS in individuals recovering from mild to moderate COVID-19 remains limited. Existing studies have primarily targeted those with severe COVID-19, where improvements in balance scores have been observed following structured rehabilitation programs [32,33].

The analysis of BBS1 scores, which reflect the ability to transition from a sitting to a standing position, revealed a statistically significant improvement following the recovery period. Although the absolute scores remained low (initial: 0.58 ± 0.96; post-recovery: 0.94 ± 1.15), the observed increase (*P* < 0.001) indicates meaningful progress in functional mobility. Despite no change in the median value (0; IQR 0–2), the upward shift in the 95^th^ percentile (from 2 to 3) suggests that a subset of participants experienced substantial gains (Table 1).

A significant improvement was also observed in BBS2 scores, which assess the ability to maintain an unsupported standing position. The mean score increased from 1.14 ± 1.28 at baseline to 1.88 ± 1.16 after recovery (*P* < 0.001), with a median change from 1 (IQR 0–3) to 2 (IQR 1–3). The difference was statistically significant and reflects a clinically relevant enhancement in postural control. The leftward skew of initial scores (with a lower quartile at 0) suggests that a considerable proportion of participants initially struggled with this task. Following recovery, the distribution shifted toward higher scores, as illustrated in the boxplot, showing improved central tendency and a reduced floor effect. These results highlight the intervention's ability to enhance static balance and upright postural stability—key components in regaining functional independence (Table 2).

The BBS3 score, which reflects the ability to maintain an unsupported sitting posture, also improved significantly following the intervention. The mean increased from 2.56 ± 1.13 to 3.23 ± 0.81 (*P* < 0.001), with a median that remained stable at three, but the interquartile range narrowed from 2–4 to 3–4. Most participants demonstrated relatively reasonable postural control at baseline, and the gains observed, though modest, were consistent and meaningful. These results suggest that, compared to the other tasks, sitting balance was initially less severely impaired but still showed positive responses to the intervention (Table 3).

When analyzing BBS1 scores specifically among women, a statistically significant improvement was observed following recovery. The mean increased from 0.65 to 1.04 points (*P* < 0.001), while the median remained stable at 0 (IQR 0–2). The percentile shift was more pronounced, with the 95^th^ percentile rising from 2 to 3 post-recovery, indicating that some individuals achieved higher performance levels. Although the central tendency remained low, these results confirm that the intervention had a positive impact on functional mobility in female participants, particularly in their ability to perform sit-to-stand transfers. The lower baseline values compared to the general sample suggest a potentially more pronounced initial deficit in this subgroup, which responded favorably to the intervention (Table 4).

In the subgroup analysis for women, BBS2 scores demonstrated a statistically significant improvement after recovery. The mean increased from 1.22 to 1.92 points (*P* < 0.001), while the median shifted from 1 (IQR 0–3) to 2 (IQR 1–3), indicating progress in the ability to maintain standing without support. The distribution of scores after recovery became more concentrated around higher values, with fewer women scoring at the lower end of the scale. This suggests that the intervention had a positive impact on postural control in standing among female participants. The results align with those observed in the total sample, reinforcing the functional relevance of the improvements noted (Table 5).

In female participants, BBS3 scores—representing balance while sitting unsupported—showed significant improvement after the intervention. The mean increased from 2.62 to 3.20 (*P* < 0.001), while the median remained at 3, and the interquartile range narrowed from 2–4 to 3–4. This pattern indicates a higher concentration of scores near the upper end of the scale following recovery. Although sitting balance was relatively preserved at baseline, the improvement signifies enhanced postural stability. These results confirm that even in tasks with less severe initial impairment, the intervention optimized motor control and postural endurance in seated conditions for female participants (Table 6).

In the male subgroup, BBS1 scores also showed a statistically significant increase following the recovery period. The mean rose from 0.48 to 0.79 (*P* = 0.001), while the median remained at 0. However, the interquartile range expanded from 0–0 to 0–2, indicating that some individuals performed better post-intervention. Although the low central tendency persisted, this improvement suggests a partial gain in the ability to initiate sit-to-stand transfers among male participants. Compared to women, men had slightly lower baseline values, and the more pronounced floor effect in the initial distribution highlights the greater functional limitation at baseline (Table 7).

In men, BBS2 scores—reflecting the ability to maintain standing without support—showed a significant increase after recovery. The mean rose from 1.03 to 1.81 (*P* < 0.001), while the median remained at 1, with the interquartile range shifting from 0–2 to 1–3. This indicates that more male participants achieved higher performance levels, although the central tendency remained moderate. The gain in standing balance suggests improved postural control, even among participants who initially exhibited significant limitations. Compared to women, the baseline values were slightly lower, but the post-recovery distribution closely aligned with the overall trend, reinforcing the intervention’s general efficacy (Table 8).

Among male participants, BBS3 scores—assessing unsupported sitting balance—significantly improved following recovery. The mean increased from 2.48 to 3.29 (*P* < 0.001), and the median rose from 2 (IQR 1–3) to 3 (IQR 3–4), indicating a substantial upward shift in performance. The narrowing of the interquartile range and the clustering of scores around higher values suggest enhanced postural stability in sitting. Compared to their baseline status, male participants showed notable functional gains in a task that already involved relatively preserved motor control, reinforcing the notion that the intervention not only prevents deterioration but can also enhance performance even in less impaired areas (Table 9).

In patients aged 60 years and older, BBS1 scores showed a statistically significant increase following recovery. The mean increased from 0.34 to 0.55 (*P* = 0.014), while the median remained unchanged at 0, and the interquartile range widened from 0–0 to 0–2. This indicates that a small proportion of participants in this age group improved their performance after the intervention, while the majority continued to face functional limitations in this task. The persistent floor effect and low central tendency suggest greater difficulty in initiating sit-to-stand transfers among older adults. Nonetheless, the statistically significant improvement supports the notion that targeted rehabilitation may yield measurable benefits even in older populations with substantial baseline limitations (Table 10).

Among patients aged 60 and older, BBS2 scores—reflecting the ability to stand unsupported—showed a statistically significant improvement after recovery. The mean increased from 0.70 to 1.46 (*P* < 0.001), with the median rising from 0 (IQR 0–1) to 1 (IQR 1–3). This upward shift indicates improved postural control in a task that many participants initially found severely limiting. Although the median remains low, the expansion of the interquartile range toward higher values suggests that a meaningful portion of older patients improved their standing balance. These results strengthen the notion that targeted rehabilitation programs can promote functional gains even in aging populations with significant baseline impairments (Table 11).

In patients aged 60 and older, BBS3 scores—representing unsupported sitting balance—significantly increased after recovery. The mean rose from 2.19 to 2.92 (*P* < 0.001), while the median shifted from 2 (IQR 1–3) to 3 (IQR 2–4). These results demonstrate a notable functional improvement in postural control during seated tasks. The narrowing of the interquartile range and the shift toward higher values indicate that many older patients responded positively to the intervention. Despite age-related limitations, these findings confirm that rehabilitation can enhance sitting balance, even in older adults with initial deficits (Table 12).

The comparison of BBS1 score differences between women and men indicated no statistically significant variation between groups (*P* = 0.425). Although the mean difference was slightly higher in women (0.39 ± 0.78) compared to men (0.32 ± 0.69), the medians were equal or similar, and the distributions were highly overlapping, as shown in Table 13. These findings suggest that the overall progression in the ability to perform sit-to-stand transfers was not influenced by gender. Therefore, both female and male participants responded similarly to the intervention regarding this specific task (Table 13).

The comparison of BBS2 score differences between female and male participants revealed no statistically significant difference between the groups (*P* = 0.263). Both genders experienced a median improvement of 1 point (IQR 0–1), and the mean scores were closely aligned: 0.70 ± 0.88 for women and 0.78 ± 0.72 for men. These findings suggest that improvements in standing balance following the intervention were not influenced by gender, supporting the general applicability of the intervention regardless of sex (Table 14).

In the case of BBS3, the comparison between genders approached statistical significance but did not reach it (*P* = 0.063). Male participants showed a slightly higher mean improvement (0.81 ± 0.84) compared to women (0.58 ± 0.84), with a median difference of one versus zero, respectively. These results indicate a similar response to the intervention across genders for this task (Table 15).

The comparison of BBS1 score changes by age revealed a statistically significant difference between groups (*P* = 0.008). Patients younger than 60 years demonstrated greater improvement (mean: 0.49 ± 0.78; median: 0, IQR 0–1) than those aged 60 years or older (mean: 0.22 ± 0.68; median: 0, IQR 0–0). These results suggest that age affects functional responses in sit-to-stand ability, with younger individuals benefiting more noticeably from the intervention. The limited progression in older adults may indicate age-related declines in neuromuscular control or greater baseline impairments (Table 16).

The analysis of BBS2 score changes by age revealed no statistically significant differences between patients younger than 60 and those aged 60 or older (*P* = 0.616). Both groups showed nearly identical median improvements (1 point, IQR 0–1), and the mean differences were also comparable: 0.71 ± 0.81 in the < 60 years and 0.76 ± 0.84 in the ≥ 60 groups. These findings suggest that age did not influence progression in standing balance, and patients from both age categories benefited similarly from the intervention for this task (Table 17).

The comparison of BBS3 score differences between age groups revealed no statistically significant difference (*P* = 0.346). The mean improvement was slightly higher in patients aged ≥ 60 years (0.73 ± 0.89) compared to those under 60 (0.61 ± 0.80), while the medians were 1 and 0, respectively, with overlapping interquartile ranges in both groups. These findings indicate that age did not significantly affect improvements in unsupported sitting balance, suggesting that younger and older participants responded similarly to the intervention in this task (Table 18).

The correlation analysis between age and BBS1 score difference revealed a statistically significant negative relationship (*P* = 0.001), with a Spearman’s rho coefficient of R = –0.267. This indicates that younger patients were more likely to improve their sit-to-stand performance after recovery. Although the strength of the association was moderate, the trend aligns with previous analyses comparing age groups. It supports the hypothesis that age is inversely associated with gains in early-phase functional mobility (Table 19 and Figure 1).

The correlation analysis between age and the BBS2 difference revealed no statistically significant association (*P* = 0.461), with a weak positive Spearman coefficient (R = 0.059). Although the regression line in Figure 2 shows a slight upward trend, the absence of significance and very low correlation strength indicate that age was not linked to changes in standing balance performance. These findings align with the earlier comparison between age groups, further supporting the notion that BBS2 improvements occurred independently of patient age (Table 20 and Figure 2).

The correlation between age and the BBS3 evolution difference was not statistically significant (*P* = 0.249), with a weak positive Spearman correlation (R = 0.092). Although the regression line in Figure 3 shows a slight upward trend, the low strength of association and the absence of statistical significance indicate that age was not significantly linked to changes in unsupported sitting balance. These results reinforce previous group comparisons and support the interpretation that improvements in BBS3 occurred regardless of patient age (Table 21 and Figure 3).

The correlation between initial BBS1 and BBS2 scores was statistically significant and strongly positive (*P* < 0.001), with a Spearman coefficient of R = 0.827. This indicates that participants who performed better in the sit-to-stand task (BBS1) also tended to score higher in the unsupported standing task (BBS2). This strong association suggests that these two tasks share overlapping functional demands, particularly in terms of lower limb strength, postural control, and balance confidence. Consequently, they may reflect a common underlying capacity for static postural transitions, especially in patients with limited initial function (Table 22 and Figure 4).

A significant positive correlation was found between initial BBS1 and BBS3 scores (*P* < 0.001, R = 0.796). Patients who scored higher on the sit-to-stand task (BBS1) also demonstrated better balance while sitting unsupported (BBS3). This association reinforces the connection between core postural control and transitional mobility. The strong correlation indicates that static seated balance may serve as a reliable indicator of early functional mobility, particularly in populations with neurological or orthopedic impairments. These findings support the internal consistency of the BBS subcomponents at the lower end of the scale (Table 23 and Figure 5).

A strong and statistically significant positive correlation was observed between initial BBS2 and BBS3 scores (*P* < 0.001, R = 0.926). Patients who performed better in unsupported standing (BBS2) also scored higher in unsupported sitting (BBS3) and vice versa. This result highlights a shared functional area between seated and standing balance, particularly in static postural control. The exceptional strength of this correlation supports the internal consistency of the early BBS subitems. It reinforces their role as reliable indicators of foundational balance capacity, especially in populations with limited motor function (Table 24 and Figure 6).

## CONCLUSION

The analysis of BBS subitem scores before and after recovery highlights consistent, statistically significant improvements across all three tasks—sit-to-stand (BBS1), unsupported standing (BBS2), and unsupported sitting (BBS3). This evolution was evident in both women and men, as well as in patients under and over 60 years of age. However, the magnitude of improvement was slightly higher in younger participants, particularly for BBS1. A weak but statistically significant negative correlation was observed between age and BBS1 evolution, while no significant associations were found between age and changes in BBS2 or BBS3 scores. Gender was not significantly associated with the magnitude of score change in any of the subitems. However, all three baseline items were highly intercorrelated, exhibiting strong, statistically significant positive correlations, particularly between BBS2 and BBS3 (R = 0.926). These results suggest that static balance tasks share standard functional foundations and that early BBS subitems are responsive to recovery-related changes while reflecting coherent aspects of postural control. Overall, the data confirm the validity and responsiveness of the BBS components in tracking short-term functional progress for patients with balance impairments, irrespective of demographic subgroups.
